# Tuberous sclerosis complex: imaging the pieces of the puzzle

**DOI:** 10.1590/0100-3984.2017.50.1e3

**Published:** 2017

**Authors:** Diana Penha

**Affiliations:** 1 Liverpool Heart and Chest Hospital NHS Foundation Trust, Liverpool, UK. E-mail: dianapenha@gmail.com.

Tuberous sclerosis complex (TSC) is a genetic syndrome that predisposes to the formation of benign tumors, commonly known as hamartomas. It affects approximately 1 in 6,000 individuals, regardless of race or ethnicity^(1)^. During the 1990s, more than 300 allelic variants of the *TSC1* gene were reported, as were more than 1,000 allelic variants of the *TSC2* gene. We now know that TSC can be inherited as an autosomal dominant disorder, although two thirds of all patients have *de novo* mutations^(2)^. In an excellent pictorial essay published in this issue of **Radiologia Brasileira**, von Ranke et al.^(3)^ review the current clinical diagnostic criteria and the radiological features of multiorgan involvement in patients with tuberous sclerosis.

The diagnosis of TSC is based on the demonstration of a mutation in the *TSC1* or *TSC2* genes^(4)^. However, in up to 25% of patients with TSC, no such mutation is identified, and the disease is known to present as a heterogeneous genetic disorder with variable clinical expression^(5)^. Regarding the difficulty of diagnosing TSC, the 2012 International Tuberous Sclerosis Complex Consensus Conference provided new recommendations that help standardize the approach to managing TSC, regardless of patient age or severity of the disease^(4)^. The recommendations state that the involvement of multiple organ systems, at different stages in life, presents major difficulties in locating and identifying the expertise to comprehensively manage the medical care of individuals with TSC. In that scenario, a detailed evaluation of the brain, kidney, lung, skin, teeth, heart and eye are crucial, and for most of these, imaging plays an important role, not only in diagnosing and determining the extent of tuberous sclerosis but also in the treatment planning and patient follow-up^(3,4,6)^.

Given the recent improvements in knowledge of TSC, as well as the recent technological advances in imaging evaluation, the pictorial review conducted by von Ranke et al.^(3)^ offers an up-to-date and valuable aid in the presumptive diagnosis and determination of the extent of TSC, informing therapeutic decision-making. In their review, the most common manifestations of TSC were systematically organized and illustrated as intracranial, pulmonary, cardiac, renal, and other (such as skin and bone abnormalities)^(6-11)^.

In recent decades, due to major advances in the field of cardiothoracic imaging, imaging features have come to be recognized as important clues to diagnosis and prognosis. The pulmonary manifestations of TSC are known not only by the presence of lymphangioleiomyomatosis (LAM), a rare entity of unknown etiology that affects women almost exclusively, with diffuse interstitial proliferation of bundles of smooth muscle cells and cystic changes, but also by the less commonly seen multifocal micronodular pneumocyte hyperplasia (MMPH)^(7,12)^. MMPH is extremely rare and may occur in isolation or in association with LAM. MMPH consists of multifocal nodular lesions related to the proliferation of type II pneumocytes, with mild thickening of the alveolar septa, particularly when extensive. The computed tomography features of MMPH include multiple bilateral ground-glass nodules. The differential diagnosis of MMPH includes other nodular/miliary conditions, such as tuberculosis, sarcoidosis, histiocytosis, "pulmonary tumorlets", and metastases^(12-14)^, as well as other diseases with a ground-glass appearance, the most important being atypical adenomatous hyperplasia and adenocarcinoma *in situ*, such diseases requiring histological confirmation^(15)^.

Another curious recent finding is the presence of focal, well-circumscribed fatty foci in the myocardium of TSC patients. Adriaensen et al.^(16)^ demonstrated foci of fat attenuation within the myocardium in 35 (64%) of 55 patients with TSC. The authors found that such foci could be single or multiple, possible locations including the interventricular septum, left ventricle wall, right ventricle wall, and papillary muscles, and ranged from 3 mm to 62 mm in size. In addition, their study raises the hypothesis that the intramyocardial fat seen in patients with TSC differentiated from perivascular epithelioid cells, having the same genetic and immunohistochemical characteristics as those giving rise to angiomyolipomas, which often accompany LAM.

A more recent study, conducted in 2015 by Tresoldi et al.^(17)^, estimated the association between myocardial fatty foci (MFF) seen on computed tomography of the chest and the type of gene mutation or multiorgan involvement in patients with TSC. Those authors found that the presence of MFF was highly specific for the disease and was associated not only with TSC gene mutations but also with brain or multiorgan involvement. The authors also stated that the number of MFF per patient correlated with the degree of multiorgan involvement.

In conclusion, an accurate diagnosis is crucial to the timely implementation of appropriate medical surveillance and treatment, as well as to determining the prognosis. Recent advances in imaging interpretation and technique have added important pieces to the puzzle of TSC diagnosis, pieces that are particularly useful in atypical clinical presentations or in cases of an inadequate therapeutic response. The pictorial review of TSC published in this issue of **Radiologia Brasileira** represents an outstanding and educative approach to the imaging evaluation of these patients, focusing on the pivotal role that imaging plays in the diagnosis, timely initiation of therapy, and prognosis of this elusive disease.
